# 7,8-dihydroxyflavone ameliorates motor deficits via regulating autophagy in MPTP-induced mouse model of Parkinson’s disease

**DOI:** 10.1038/s41420-021-00643-5

**Published:** 2021-09-20

**Authors:** Li Zuo, Chunfang Dai, Lilin Yi, Zhifang Dong

**Affiliations:** Pediatric Research Institute, Ministry of Education Key Laboratory of Child Development and Disorders, National Clinical Research Center for Child Health and Disorders, China International Science and Technology Cooperation Base of Child Development and Critical Disorders, Chongqing Key Laboratory of Translational Medical Research in Cognitive Development and Learning and Memory Disorders, Children’s Hospital of Chongqing Medical University, Chongqing, 400014 China

**Keywords:** Parkinson's disease, Parkinson's disease

## Abstract

Parkinson’s disease (PD) is a neurodegenerative disease characterized by the loss of dopaminergic neurons in the substantia nigra and diminished dopamine content in the striatum. Recent reports show that 7,8-dihydroxyflavone (DHF), a TrkB agonist, attenuates the α-synuclein deposition and ameliorates motor deficits. However, the underlying mechanism is unclear. In this study, we investigated whether autophagy is involved in the clearance of α-synuclein and the signaling pathway through which DHF exerts therapeutic effects. We found that the administration of DHF (5 mg/kg/day, i.p.) prevented the loss of dopaminergic neurons and improved motor functions in the 1-methyl-4-phenyl-1,2,3,6-tetrahydropyridine (MPTP) mouse model of PD, whereas these protective effects of DHF were completely blocked by autophagy inhibitor chloroquine (CQ). Further in vitro studies showed that autophagy was inhibited in N2A cells treated with 1-methyl-4-phenylpyridinium (MPP^+^), as reflected by a significant decrease in the expressions of autophagy marker proteins (Beclin1 and LC3II) and an increase in the expression of autophagic flux marker p62. DHF restored the impaired autophagy to control level in MPP^+^-treated N2A cells by inhibiting the ERK-LKB1-AMPK signaling pathway. Taken together, these results demonstrate that DHF exerts therapeutic effects in MPTP/MPP^+^-induced neurotoxicity by inhibiting the ERK-LKB1-AMPK signaling pathway and subsequently improving impaired autophagy.

## Introduction

Parkinson’s disease (PD) is a neurodegenerative disorder with high incidence that only second to Alzheimer’s disease. The main pathological changes of PD are the loss of dopaminergic neurons and deposition of intraneuronal inclusions known as Lewy bodies [1]. Even though the pathogenesis of PD has not been fully clarified, accumulating studies have shown that various intracellular processes are involved in it, including misfolding and aggregation of α-synuclein, endoplasmic reticulum stress, mitochondrial dysfunction, dysregulated calcium homeostasis, and dysfunctional autophagy [2, 3].

Especially, autophagy has been drawn a lot of attention in recent years. It has been reported that impaired autophagy exists in the brains of both PD patients and PD animal models [4–6]. Animal models with disrupted autophagy-lysosome system caused by deletion of essential autophagy gene are more likely to show PD-like pathology, which further confirmed that autophagy plays a neuroprotective role during the pathogenesis of PD [6, 7]. As a crucial determinant of neurotoxicity, the level of α-synuclein is confirmed to be closely related to autophagy in recent studies [8]. The degradation of α-synuclein was majorly dependent on ALP (autolysosome pathway) and UPS (ubiquitin-proteasome pathway) [9]. It has been reported that the degradation capacity of UPS is reduced in aged animals [10], resulting in α-synuclein aggregation and consequently contributing to PD pathogenesis. However, autophagy may compensate for the decreased degradation mediated by UPS [9, 10]. Therefore, promoting autophagy through pharmacological or other methods to increase the degradation of α-synuclein may be a potential therapeutic target for PD [11].

Brain-derived neurotrophic factor (BDNF) can regulate a variety of biological processes in human body, especially in neurological system. It binds to the TrkB receptor and triggers dimerization and autophosphorylation of tyrosine residues in the intracellular domain, resulting in activation of signaling pathways including mitogen-activated protein kinase (MAPK), phosphatidylinositol 3-kinase (PI3K) and phospholipase-γ (PLC-γ) [12]. BDNF displays therapeutic effects in numerous neurological, mental and metabolic disorders [13, 14]. However, the use of recombinant BDNF has turned out to be disappointingly useless in clinical trials, probably due to poor drug delivery to the brain, short half-life period, and other factors that restrict its clinical use [15, 16]. It is exciting that 7,8-dihydroxyflavone (DHF) is screened from thousands of compounds, which is orally bioavailable and easily penetrates the brain-blood barrier (BBB) to exert its neurotrophic effects in the central nervous system (CNS) [12, 17, 18]. Importantly, compared with BDNF, DHF has better clinical application characteristics. For instance, BDNF activated TrkB receptor and elicited receptor ubiquitination and degradation, while DHF does not induce TrkB ubiquitination or degradation [19]. In addition, DHF specifically acts on TrkB receptor, but BDNF also binds to p75NTR that could mediate the cell death pathway [20], apart from the TrkB receptor. Several studies have shown that treatment with DHF prevents α-synuclein accumulation and loss of dopaminergic neurons as well as ameliorating motor deficits in the MPTP-induced PD mouse model [12, 21, 22]. Consistent with that, our recent study shows that DHF treatment suppresses α-synuclein accumulation and reduces oxidative stress, and subsequently blocks dopaminergic neuron loss in the substantia nigra (SN) and striatum [23]. However, the mechanism of DHF reducing the α-synuclein accumulation remains largely unclear. As forementioned, the accumulation of α-synuclein was closely connected with impaired autophagy. Previous studies have shown that BDNF-TrkB promotes autophagy flux [24, 25]. In addition, DHF binds to TrkB receptor and regulates the downstream signaling cascade including PI3K-AKT, MAPK, which play a role in the regulation of autophagy [26, 27]. We, therefore, supposed that DHF may reduce α-synuclein aggregation via promoting autophagy. In the present study, we investigated this hypothesis by using a combination of biochemical, pharmacological, and behavioral assessments in PD models of both in vivo and in vitro.

## Results

### Autophagy inhibitor chloroquine abrogates the protective effects of DHF on motor deficits in MPTP-induced mouse model of PD

Alterations in the function of autophagy to degrade misfolded and aggregated proteins or to eliminate damaged mitochondria are being recognized as an important factor in the pathogenesis of PD [28, 29]. Our recent study has shown that DHF treatment ameliorates MPTP-induced motor deficits and reduces the loss of dopaminergic neurons in mice [23]. To determine whether autophagy is involved in these effects, we introduced autophagy inhibitor chloroquine in the present study. Three behavioral tests were conducted: rotarod test, pole test, and wire suspension test.

**Fig. 1:**
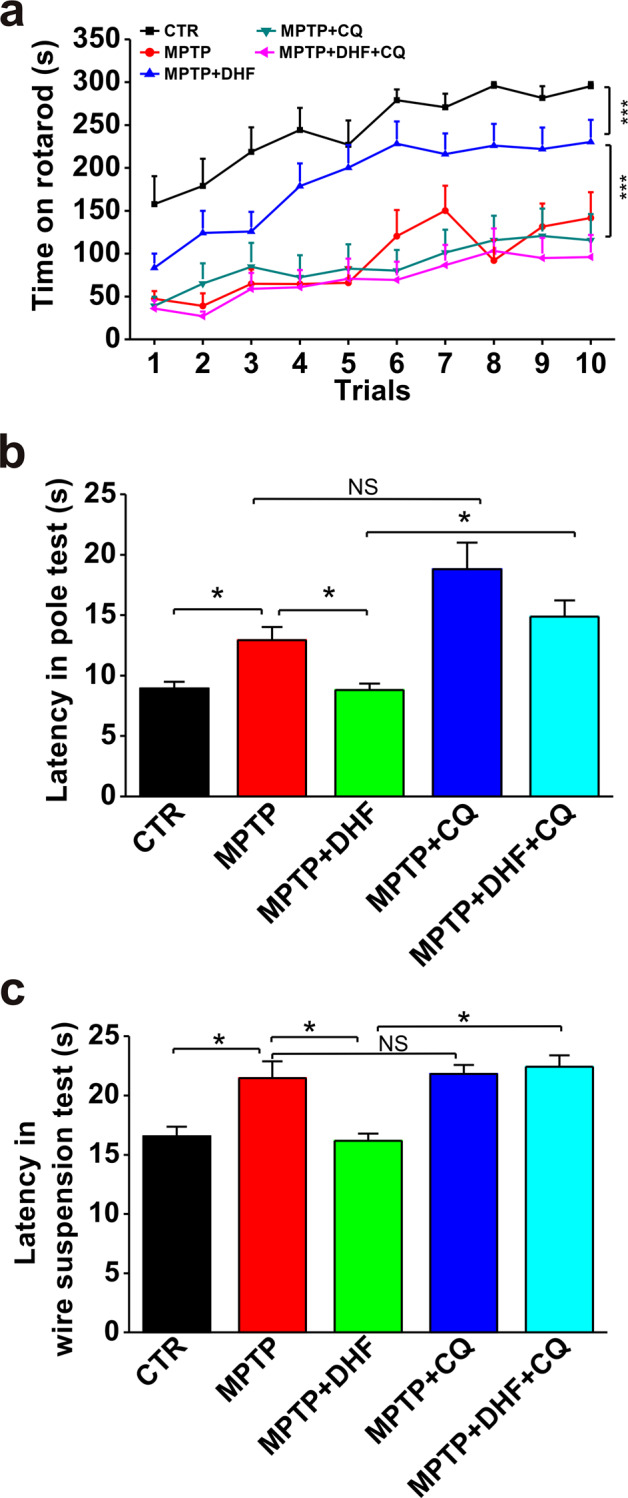
Chloroquine (CQ) prevents the protective effects of DHF on motor deficits in MPTP-induced mouse model of PD. **a** Latency to fall off the rod in the rotarod test was significantly reduced by MPTP administration, and DHF treatment restored the latency to control level. CQ abolished the recovery of DHF in MPTP-induced PD mouse. **b** Latency to descend in the pole test was markedly increased by MPTP administration and DHF treatment restored the latency to control level. CQ abrogated the recovery of DHF in MPTP-induced PD mouse. **c** Latency to reach the platform in the wire suspension test was significantly increased by MPTP administration, and DHF treatment restored the latency to control level while CQ abolished the recovery of DHF in MPTP-induced PD. Data are expressed as mean ± SEM. *P < 0.05, ***P < 0.001.

In rotarod test, mice in MPTP-treated group spent significantly less time on the rod compared with controls (CTR), and DHF treatment (5 mg/kg, i.p.) prolonged the latency on the rod. However, the beneficial effect of DHF was diminished by autophagy inhibitor chloroquine (CQ) treatment, as reflected by the latency decreasing to the same level of MPTP (CTR: *n* = 15; MPTP: *n* = 19; MPTP + DHF: *n* = 19; MPTP + DHF + CQ: *n* = 22; MPTP + CQ: *n* = 15; Fig. 1a). In pole test, the mice in MPTP group spent much more time on descending to the floor compared with CTR, and DHF treatment significantly shortened the descending time. Similar to the findings in rotarod test, chloroquine markedly increased the descending time, indicating it abrogated the protective effect of DHF (CTR: *n* = 15, 8.9 ± 0.5 s; MPTP: *n* = 19, 12.9 ± 1.0 s, *P* < 0.05 vs. CTR; MPTP + DHF: *n* = 19, 8.8 ± 0.5 s, *P* > 0.05 vs. CTR, *P* < 0.05 vs. MPTP; MPTP + DHF + CQ: *n* = 22, 14.8 ± 1.3 s, *P* < 0.001 vs. MPTP + DHF; MPTP + CQ: *n* = 15, 18.8 ± 2.2 s, *P* > 0.05 vs. MPTP; Fig. 1b). Finally, in wire suspension test, DHF treatment shortened the time to reach the platform in MPTP-treated mice, while chloroquine treatment made it back to MPTP group level (CTR: *n* = 15, 16.5 ± 0.8 s; MPTP: *n* = 19, 21.4 ± 1.4 s, *P* < 0.01 vs. CTR; MPTP + DHF: *n* = 19, 16.1 ± 0.6 s, *P* > 0.05 vs. CTR, *P* < 0.05 vs. MPTP; MPTP + DHF + CQ: *n* = 22, 22.4 ± 0.9 s, *P* < 0.001 vs. MPTP + DHF; MPTP + CQ: *n* = 15, 21.8 ± 0.7 s, *P* > 0.05 vs. MPTP; Fig. 1c). Notably, chloroquine alone had no influence on motor functions in MPTP-treated mice (Fig. 1a–c). Together, these results suggest that DHF rescued the motor deficits in MPTP-induced PD mice, while chloroquine prevents the protective effects of DHF.

### Chloroquine prevents the effects of DHF on the restoration of TH and the decrease of α-synuclein accumulation

**Fig. 2:**
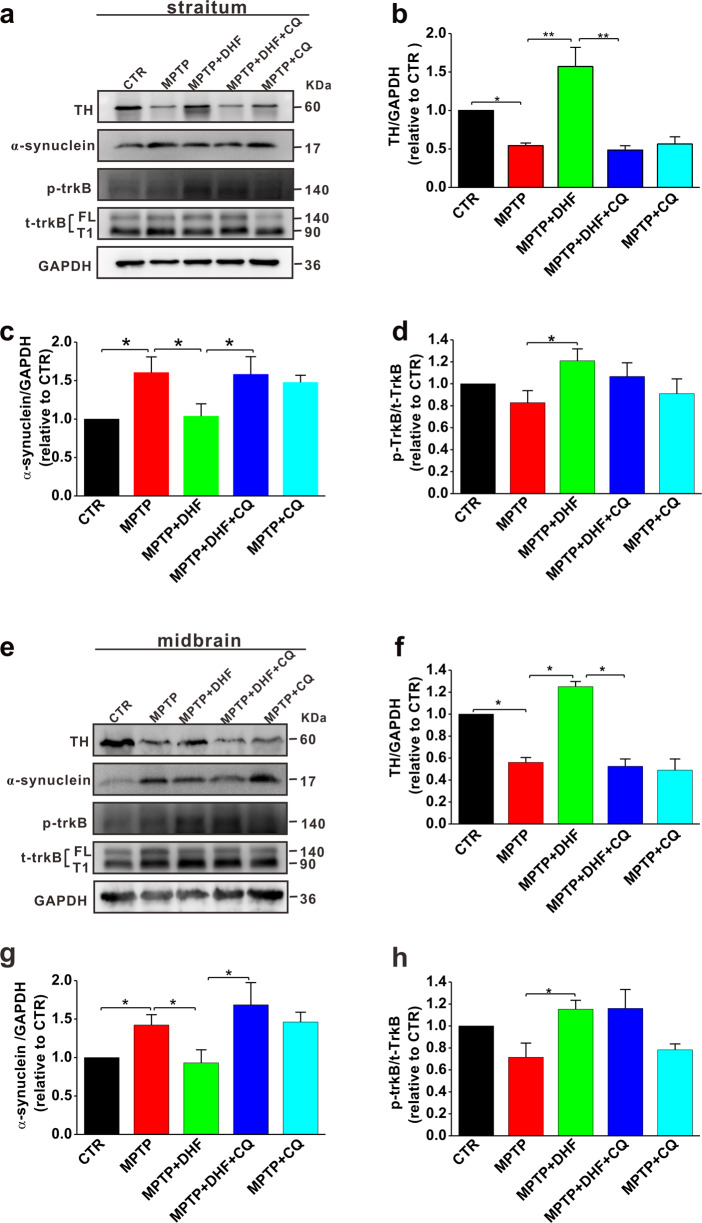
Chloroquine (CQ) prevents the DHF-induced restoration of TH and decrease of α-synuclein accumulation in MPTP-induced mouse model of PD. **a**–**d** The protein levels of TH (**b**), α-synuclein (**c**), and p-TrkB (**d**) assessed by western blot in the striatum from MPTP-induced mouse model of PD. **e**–**h** The protein levels of TH (**f**), α-synuclein (**g**) and p-TrkB (**h**) assessed by western blot in the midbrain from MPTP-induced mouse model of PD. Data are expressed as mean ± SEM. *P < 0.05, **P < 0.01.

The accumulation of α-synuclein and the lack of tyrosine hydroxylase (TH), a rate-limiting enzyme during biosynthesis of L-dihydroxyphenylalanine (L-DOPA), are thought to contribute to PD progression [30]. Therefore, we next examined the α-synuclein and TH expression in the striatum and midbrain in MPTP-induced PD model mice with or without chloroquine treatment. The results showed that the MPTP administration significantly increased α-synuclein accumulation and decreased TH expression in both the striatum (α-synuclein: n = 5 in each group; MPTP: 1.6 ± 0.2 relative to CTR, *P* < 0.05 vs. CTR; TH: *n* = 5 in each group; MPTP: 0.54 ± 0.03 relative to CTR, *P* < 0.01 vs. CTR; Fig. 2a–c) and midbrain (α-synuclein: *n* = 5 in each group; MPTP: 1.52 ± 0.06 relative to CTR, *P* < 0.01 vs. CTR; TH: *n* = 5 in each group; MPTP: 0.56 ± 0.04 relative to CTR, *P* < 0.01 vs. CTR; Fig. 2e–g), compared with those in control group. Consistent with our recent report [23], DHF treatment restored α-synuclein accumulation and TH expression to control levels (α-synuclein: MPTP + DHF: 1.03 ± 0.06 relative to CTR, *P* < 0.05 vs. MPTP; TH: MPTP + DHF: 1.57 ± 0.25 relative to CTR, *P* < 0.05 vs. MPTP Fig. 2a–c, e–g). However, the effects of DHF on increasing TH expression and reducing α-synuclein accumulation were completely prevented by chloroquine treatment (α-synuclein: MPTP + DHF + CQ: 1.58 ± 0.22 relative to CTR, *P* < 0.05 vs. MPTP; TH: MPTP + DHF + CQ: 0.48 ± 0.06 relative to CTR, *P* < 0.01 vs. MPTP; Fig. 2a–c, e–g). As DHF binds to the TrkB extracellular domain and promotes its dimerization and autophosphorylation at tyrosine residues (Y705/706), we next tested the expression of total TrkB (t-TrkB), including full-length isoform (TrkB-FL) and a truncated protein lacking the tyrosine-kinase domain that is strikingly similar to the inactive TrkB-T1 isoform (TrkB-T1), and corresponding phosphorylated TrkB (p-TrkB). The results showed that DHF treatment increased p-TrkB level in both the striatum (*n* = 5 in each group; MPTP: 0.82 ± 0.1 relative to CTR, *P* < 0.05 vs. CTR; MPTP + DHF: 1.21 ± 0.1 relative to CTR, *P* < 0.05 vs. CTR, *P* < 0.05 vs. MPTP; Fig. 2a, d) and midbrain (CTR: *n* = 5 in each group; MPTP: 0.71 ± 0.12 relative CTR, *P* < 0.05 vs. CTR; MPTP + DHF: 1.15 ± 0.08 relative to CTR, *P* < 0.05 vs. CTR, *P* < 0.05 vs. MPTP; Fig. 2e, h), compared with those in MPTP-treated mice. Notably, chloroquine treatment displayed no effect on p-TrkB expression (Fig. 2a, d, e, h).

### Chloroquine prevents the neuroprotective effects of DHF on dopaminergic neurons

**Fig. 3:**
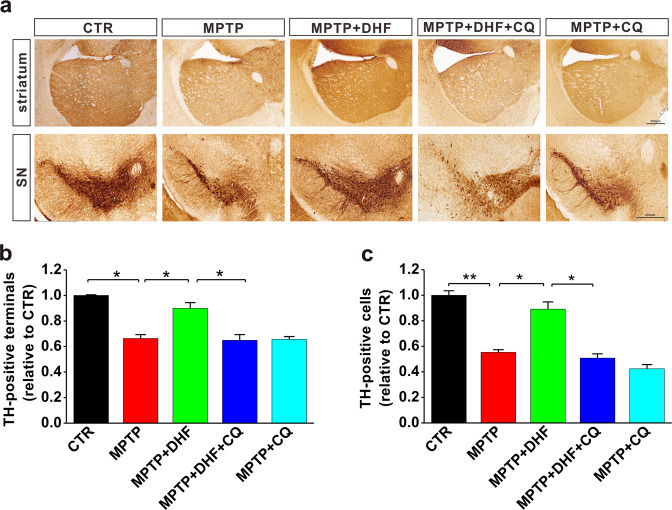
Chloroquine (CQ) prevents the protective effects of DHF on the loss of dopaminergic neurons in MPTP-induced mouse model of PD. **a** Representative photomicrographs of TH-positive terminals in striatum and TH-positive neurons in midbrain. **b**, **c** Quantitative analysis of TH-positive terminals in the striatum (**b**) and TH-positive cells in the midbrain (**c**). Data are expressed as mean ± SEM. *P < 0.05, **P < 0.01.

To further confirm whether autophagy is involved in protective effects of DHF on dopaminergic neurons after MPTP insult, immunohistochemistry assay was used to examine the TH-positive neurons in the SN and terminals in the striatum. Results showed that the number of TH-positive neurons in the SN was significantly decreased after MPTP treatment. The administration of DHF markedly blocked the loss of TH-positive neurons, while its protective effect was completely prevented by chloroquine treatment (*n* = 4 in each group; MPTP: 0.55 ± 0.02 relative to CTR, *P* < 0.05 vs. CTR; MPTP + DHF: 0.89 ± 0.05 relative to CTR, *P* > 0.05 vs. CTR, *P* < 0.05 vs. MPTP; MPTP + DHF + CQ: 0.51 ± 0.03 relative to CTR, *P* < 0.05 vs. MPTP + DHF; Fig. 3a, c). Similarly, DHF treatment increased TH-positive terminals in the striatum of MPTP-treated mice, while chloroquine blocked these changes (*n* = 4 in each group; MPTP: 0.66 ± 0.02 relative to CTR, *P* < 0.05 vs. CTR; MPTP + DHF: 0.90 ± 0.04 relative to CTR, *P* > 0.05 vs. CTR, *P* < 0.05 vs. MPTP; MPTP + DHF + CQ: 0.65 ± 0.04 relative to CTR, *P* < 0.05 vs. MPTP + DHF; Fig. 3a, b).

### DHF restores impaired autophagy in cell model of PD

**Fig. 4:**
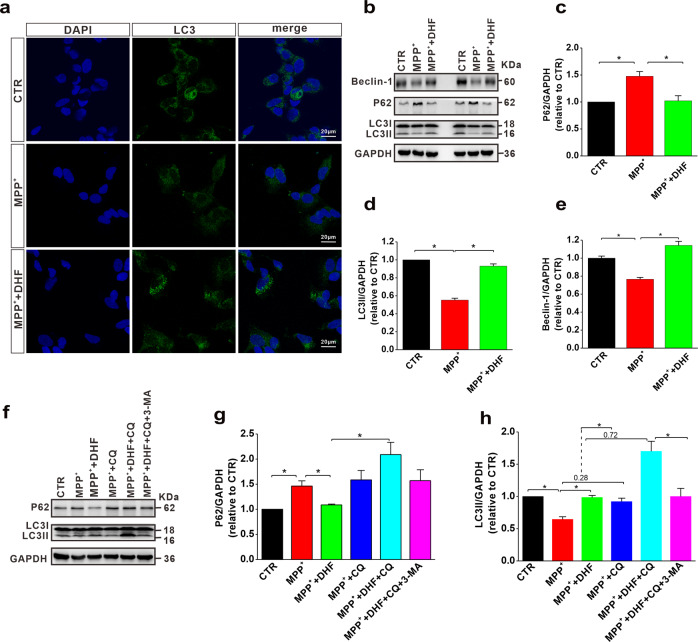
DHF induces autophagy in MPP^+^-treated N2A cells. **a** Immunofluorescence analysis of LC3 to evaluate autophagy in N2A cells. **b**–**e** The protein levels of P62 (**c**), LC3 (**d**), and Beclin-1 (**e**) assessed by western blot in N2A cells subjected to MPP^+^ treatment with or without DHF. **f**–**h** The protein levels of P62 (**g**) and LC3 (**h**) assessed by western blot in MPP^+^-treated N2A cells with or without co-administration of DHF and CQ or 3-MA. Data are expressed as mean ± SEM. *P < 0.05.

Aforementioned results indicate that DHF exerts beneficial effects on motor function and neuropathology via autophagy in MPTP-induced mouse model of PD. To further investigate the mechanism of autophagy in DHF-medicated therapeutic effects in PD, we next introduced N2A cells treated with MPP^+^, a cell model of PD. The results from immunofluorescence experiments showed that LC3 puncta was significantly increased following DHF treatment in MPP^+^-treated cells, indicating that DHF increased the formation of autophagosomes (Fig. 4a). We further examined the expression of autophagy-related proteins, including beclin-1, P62 and LC3 that are commonly used to detect autophagy [31]. The results showed that the LC3II and beclin-1 were decreased, and the expression of P62 was increased in MPP^+^ group, while DHF treatment restored the expression of P62, LC3II and beclin-1 to control levels, indicating that DHF rescued the MPP^+^-induced impairment of autophagy (P62: *n* = 6 in each group; MPP^+^: 1.47 ± 0.08 relative to CTR, *P* < 0.05 vs. CTR; MPP^+^ + DHF: 1.02 ± 0.09 relative to CTR, *P* > 0.05 vs. CTR, *P* < 0.05 vs. MPP^+^; LC3II: *n* = 6 in each group; MPP^+^: 0.55 ± 0.02 relative to CTR, *P* < 0.05 vs. CTR, MPP^+^ + DHF: 0.93 ± 0.02 relative to CTR, *P* > 0.05 vs. CTR, *P* < 0.05 vs. MPP^+^; beclin-1: *n* = 4 in each group; MPP^+^: 0.77 ± 0.02 relative to CTR, *P* < 0.05 vs. CTR, MPP^+^ + DHF: 1.14 ± 0.04 relative to CTR, *P* > 0.05 vs. CTR, *P* < 0.05 vs. MPP^+^; Fig. 4b–e). Autophagic flux is the entire process including autophagosome formation, delivery and fusion to lysosomes and subsequent degradation, which can be quantified by measuring the difference in the amount of LC3II in the presence and absence of saturating levels of inhibitors. To further determine the effects of DHF on autophagic flux, we tested the LC3II and P62 levels with or without the presence of chloroquine, an autophagy inhibitor that neutralizes lysosome PH [32]. We observed an accumulation of P62 after MPP^+^ treatment, which suggests that autophagic flux is blocked, while DHF treatment repaired this interrupted autophagic flux as reflected by decreased P62 (*n* = 4 in each group; MPP^+^: 1.46 ± 0.10 relative to CTR, *P* < 0.05 vs. CTR; MPP^+^ + DHF: 1.09 ± 0.02 relative to CTR, *P* > 0.05 vs. CTR, *P* < 0.05 vs. MPP^+^; Fig. 4b, c). As expected, P62 levels were increased in all the CQ treated groups compared with CTR and MPP^+^ + DHF (*n* = 4 in each group; MPP^+^ + CQ: 1.59 ± 0.19 relative to CTR, *P* < 0.05 vs. CTR, *P* < 0.05 vs. MPP^+^ + DHF; MPP^+^ + DHF + CQ: 2.09 ± 0.25 relative to CTR, *P* < 0.05 vs. CTR, *P* < 0.05 vs. MPP^+^ + DHF; MPP^+^ + DHF + CQ + 3-MA: 1.57 ± 0.22 relative to CTR, *P* < 0.05 vs. CTR, *P* < 0.05 vs. MPP^+^ + DHF; Fig. 4f, g), which suggests CQ interrupted autophagy. We found that CQ administration increased LC3II levels in both the MPP^+^ and MPP^+^ + DHF groups, but the magnitude of the increase was significantly lower in the MPP^+^ group (the difference between MPP^+^ and MPP^+^ + CQ was 0.28) than in MPP^+^ + DHF group (the difference between MPP^+^ + DHF and MPP^+^ + DHF + CQ was 0.72) (*n* = 4 in each group; Fig. 4f, h), which suggests that DHF increased autophagic flux. In addition, 3-MA, an autophagy inhibitor that blocks the early stage of autophagy by inhibiting class III PI3K, reduced the accumulation of LC3II in MPP^+^ + DHF group caused by chloroquine (*n* = 4 in each group; MPP^+^ + CQ: 0.91 ± 0.05, relative to CTR, MPP^+^ + DHF + CQ: 1.7 ± 0.16 relative to CTR, *P* < 0.05 vs. MPP^+^ + CQ; MPP^+^ + DHF + CQ + 3-MA: 1.0 ± 0.12 relative to CTR, *P* < 0.05 vs. MPP^+^ + DHF + CQ; Fig. 4f, h), suggesting an increased autophagosome formation.

### ANA-12 blocked the DHF-induced autophagy in PD cell model

**Fig. 5:**
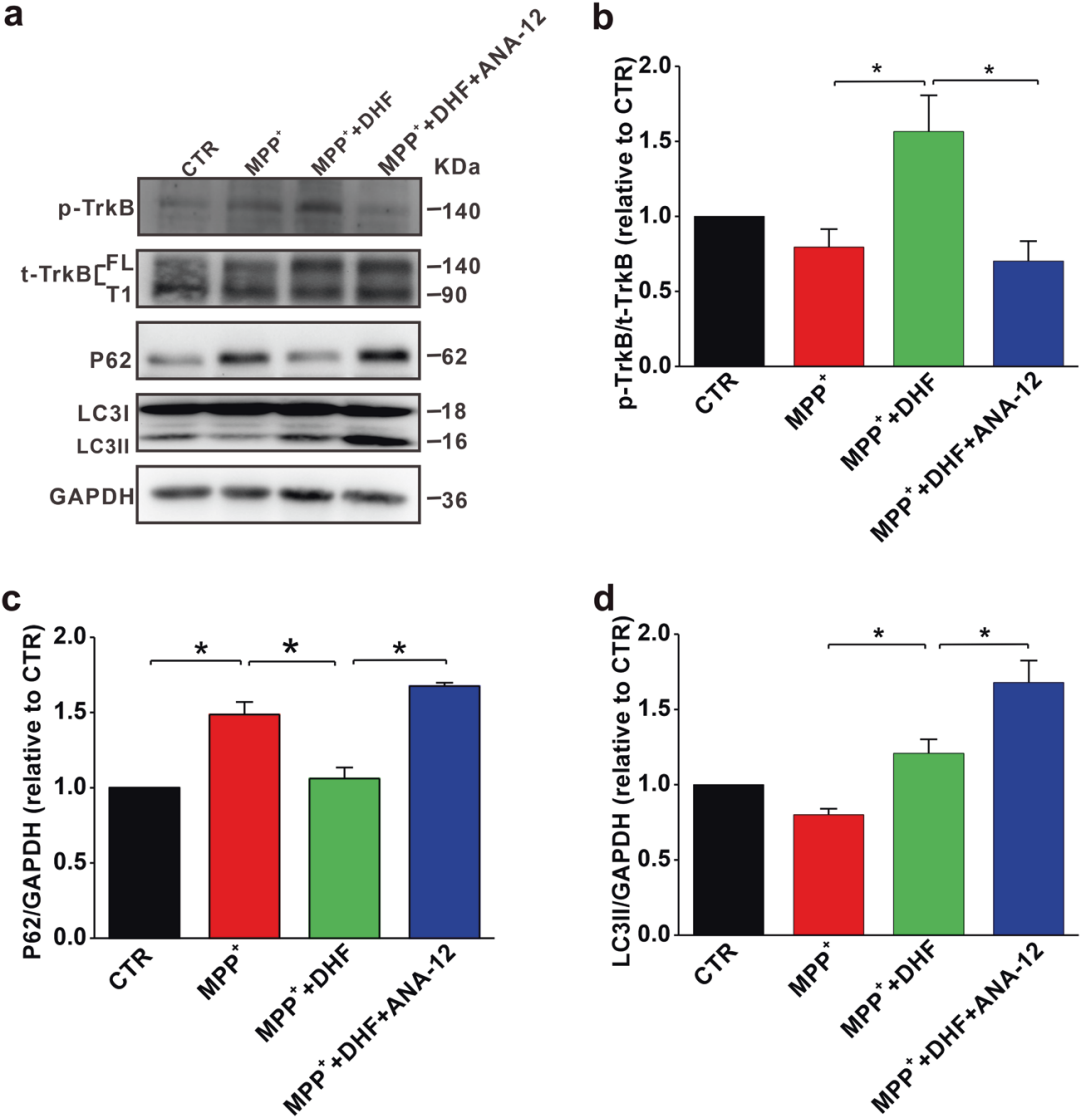
TrkB inhibitor ANA-12 blocks the DHF-induced autophagy. **a** Sequential immunoblotting of cell lysates. **b**–**d** The protein levels of p-TrkB (**b**), P62 (**c**) and LC3 (**d**) assessed by western blot in MPP^+^-treated N2A cells with or without co-administration of DHF and ANA-12. Data are expressed as mean ± SEM. *P < 0.05.

We have found that DHF treatment increases TrkB phosphorylation (Fig. 2) and autophagy (Fig. 4) in PD models. To determine whether the DHF-induced autophagy was dependent on TrkB activation, a specific TrkB antagonist ANA-12 was introduced. The results showed that ANA-12 completely blocked the DHF-induced increase of p-TrkB in MPP^+^-treated N2A cells (*n* = 4 in each group; MPP^+^: 0.80 ± 0.12 CTR, *P* < 0.05 vs. CTR; MPP^+^ + DHF: 1.56 ± 0.24 relative to CTR, *P* < 0.05 vs. CTR, *P* < 0.05 vs. MPP^+^; MPP^+^ + DHF + ANA-12: 0.70 ± 0.13 relative to CTR, *P* < 0.05 vs. CTR, *P* < 0.05 vs. MPP^+^ + DHF; Fig. 5a, b). No significant difference was found in total TrkB expression (Fig. 5a, b). We also found that the DHF-induced autophagy was abrogated by ANA-12, as the expression of both P62 and LC3II were increased, indicating autophagosomes were accumulated and autophagy pathway was interrupted in late stage (P62: *n* = 4 in each group; MPP^+^: 1.48 ± 0.08 relative to CTR, *P* < 0.05 vs. CTR; MPP^+^ + DHF: *n* = 4, 1.06 ± 0.07 relative to CTR, *P* > 0.05 vs. CTR, *P* < 0.05 vs. MPP^+^; MPP^+^ + DHF + ANA-12: 1.67 ± 0.02 relative to CTR, *P* < 0.05 vs. MPP^+^ + DHF; LC3II: *n* = 4 in each group; MPP^+^: 0.80 ± 0.04 relative to CTR, *P* < 0.05 vs. CTR; MPP^+^ + DHF: 1.20 ± 0.09 relative to CTR, *P* > 0.05 vs. CTR; *P* < 0.05 vs. MPP^+^; MPP^+^ + DHF + ANA-12: 1.67 ± 0.14 CTR, *P* < 0.05 vs. MPP^+^ + DHF; Fig. 5a, c, d). These results suggest that the DHF-induced autophagy may be executed through binding to TrkB receptors.

### DHF promotes autophagy through ERK-LKB1-AMPK pathway

**Fig. 6:**
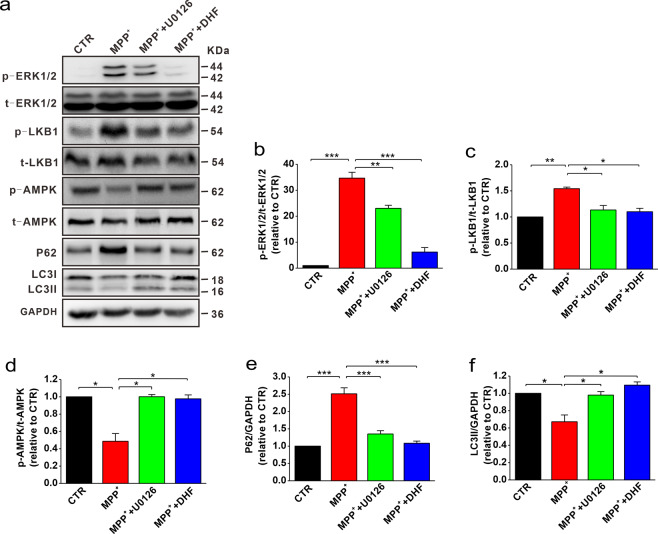
DHF-promoted autophagy is dependent on ERK-LKB1-AMPK pathway. **a** Sequential immunoblotting of cell lysates. **b**–**f** The protein levels of p-ERK1/2 (**b**), p-LKB1 (**c**), p-AMPK (**d**), P62 (**e**) and LC3 (**f**) assessed by western blot in MPP^+^-treated N2A cells with or without co-administration of DHF and U0126. Data are expressed as mean ± SEM. *P < 0.05, **P < 0.01, ***P < 0.001.

Previous studies have shown that DHF binds to TrkB receptor and activates PI3K/Akt, MAPK, and PLC-γ signaling cascades [17]. Therefore, we next wanted to examine the downstream signal pathways that may be involved in DHF-mediated autophagy. Consistent with previous report [33], we found that the expression of phosphorylated ERK1/2 (p-ERK1/2) was significantly increased in MPP^+^-treated cells compared with control, while DHF treatment restored the p-ERK1/2 expression to control level (*n* = 5 in each group; MPP^+^: 34.7 ± 2.25 relative to CTR, *P* < 0.001 vs. CTR; MPP^+^ + DHF: 6.17 ± 0.02 relative to CTR, *P* < 0.001 vs. MPP^+^; Fig. 6a, b). To further determine whether ERK1/2 played a critical role in autophagy, we introduced ERK1/2 inhibitor U0126 and measured the expression of P62 and LC3II. The results showed that U0126 treatment following MPP^+^ markedly reduced p-ERK1/2 expression (*n* = 5 in each group; MPP^+^: 34.7 ± 2.25 relative to CTR, *P* < 0.001 vs. CTR; MPP^+^ + U0126: 23.0 ± 1.1 relative to CTR, *P* < 0.05 vs. MPP^+^; Fig. 6a, b), concomitantly with increased LC3II and decreased P62 (LC3II: *n* = 5 in each group; MPP^+^: 0.67 ± 0.08 relative to CTR, *P* < 0.001 vs. CTR; MPP^+^ + U0126: 0.98 ± 0.04 relative to CTR, *P* < 0.05 vs. MPP^+^; MPP^+^ + DHF: 1.09 ± 0.04 relative to CTR, *P* < 0.001 vs. MPP^+^; P62: *n* = 5 in each group; MPP^+^: 2.51 ± 0.17 relative to CTR, *P* < 0.001 vs. CTR; MPP^+^ + U0126: 1.35 ± 0.10 relative to CTR, *P* < 0.05 vs. MPP^+^; MPP^+^ + DHF: 1.08 ± 0.06 relative to CTR, *P* < 0.001 vs. MPP^+^; Fig. 6a, e, f), indicating that suppressing ERK1/2 activation induced autophagy.

Since AMPK and mTOR pathway play an important role in the regulation of autophagy, we then investigated whether AMPK and mTORC1 were involved in DHF-induced autophagy. The results showed that the phosphorylated AMPK (p-AMPK) was significantly decreased in MPP^+^-treated cells (*n* = 5 in each group; MPP^+^: 0.49 ± 0.09 relative to CTR, *P* < 0.05 vs. CTR; Fig. 6a, d), while p-mTOR remained unchanged (*n* = 4 in each group; Fig. S1a, c). DHF treatment increased p-AMPK to control level (*n* = 5 in each group; MPP^+^: 0.49 ± 0.09 relative to CTR, MPP^+^ + DHF: 0.98 ± 0.05 relative to CTR, *P* < 0.05 vs. MPP^+^; Fig. 6a, d). As the suppression of ERK1/2 activation induced autophagy, we next wanted to determine whether there is a link between p-ERK1/2 and p-AMPK. The results showed that p-AMPK (Thr172) was increased after treatment with ERK1/2 inhibitor U0126, indicating ERK1/2 may negatively regulated AMPK phosphorylation (MPP^+^: 0.49 ± 0.09 relative to CTR, MPP^+^ + U0126: 1.0 ± 0.02 relative to CTR, *P* < 0.05 vs. MPP^+^; Fig. 6a, d). The phosphorylation of AMPK can be regulated by constitutively active kinase STK11/LKB1 [34]. The previous study has shown that ERK1/2 can inhibit LKB1 through phosphorylation of Ser428 residue, and LKB1, in turn, acts upstream of the AMPK/ULK1/ATG1 signal axis to regulate autophagy [34, 35]. We, therefore, tested the expression of p-LKB1 (Ser428). In agreement with previous study, DHF or U0126 attenuated the level of p-ERK1/2 and p-LKB1 (*n* = 5 in each group; MPP^+^: 1.54 ± 0.03 relative to CTR, *P* < 0.05 vs. CTR; MPP^+^ + U0126: 1.13 ± 0.08 relative to CTR, *P* < 0.05 vs. MPP^+^; MPP^+^ + DHF: 1.10 ± 0.065 relative to CTR, *P* < 0.05 vs. MPP^+^; Fig. 6a, c, d). Alternatively, AMPK (Thr172) is also phosphorylated by the Ca^2+^-activated protein kinase CaMKK2/CaMKKβ [36], which can be activated by TrkB downstream PLC-γ-IP3-Ca^2+^ signaling. However, we found that PLC-γ inhibitor U73122 had no effect on DHF-induced autophagy, suggesting that PLC-γ may not involve in this process (Fig. S2). Together, these findings suggest that DHF decreased ERK1/2 phosphorylation, and subsequently regulated LKB1 and AMPK activation, thereby promotes autophagy in PD models.

## Discussion

**Fig. 7:**
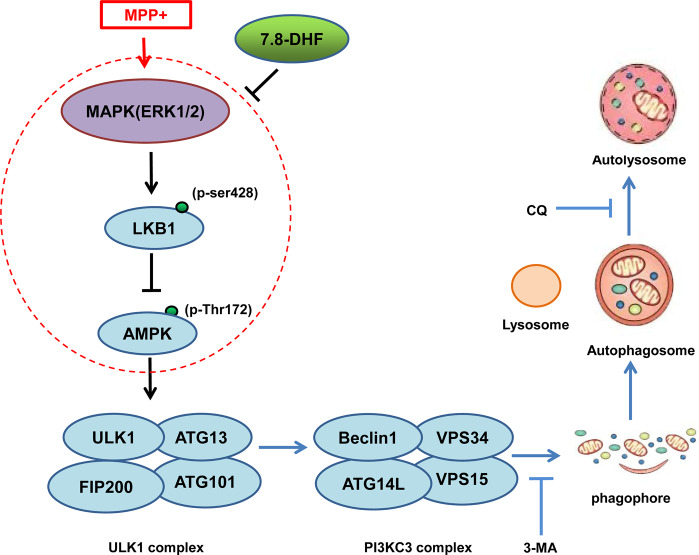
Proposed model for DHF-improved autophagy in PD. ERK1/2 was highly phosphorylated (p-ERK1/2) in MPP^+^-treated PD model cells, which led to LKB1 phosphorylation at Ser428 and impaired the ability of LKB1 to phosphorylate AMPK at Thr172, thereby blocking the activation of AMPK and AMPK-initiated autophagy. DHF inhibited the ERK1/2 activation and restored the ability of LKB1 in activating AMPK and autophagy.

In the present study, we first confirm that DHF has protective effects on motor function and neuropathology in MPTP-induced PD model mice, and also demonstrate that DHF exerts protective effects by restoring impaired autophagy through ERK-LKB1-AMPK pathway (Fig. 7).

As a mimetic of BDNF, DHF has been gained a lot of attention as a therapeutic target in various BDNF-implicated human diseases, especially in neurological disorders. In previous studies, the protective effects of DHF were mainly focus on its anti-apoptosis, anti-inflammatory, and anti-oxidative function [37, 38]. It has been well documented that autophagy is a biological process that requires a sequential stimulation of molecular events including autophagosome formation, delivery to the lysosome and eventual fusion with lysosomes to degradation [39]. The impairment of autophagy is thought to contribute to the pathogenesis of many neurodegenerative diseases including PD [4–6]. Consistently, we here have reported that the autophagy is impaired in PD models, and importantly, we have found that DHF induces autophagy and enhances α-synuclein degradation through autolysosome pathway (Fig. 4). This finding is supported by a recent study that DHF improves autophagy and alleviates the depressive behaviors in animal models [40]. The Atg1/ULK1 is a central component and plays a crucial role in the process of autophagy especially in the autophagosome formation during which ULK1, ATG13, ATG101, and FIP200 assemble into ULK1 complex and regulate autophagosome formation [41]. ULK1 is mainly regulated by mTOR and AMPK [42]. In the present study, we have found that DHF increases AMPK phosphorylation at Thr172, and then increases phosphorylation of ULK1 at Ser555 (Fig. S1a, b), thereby increases autophagy in PD.

DHF promotes TrkB receptor dimerization and autophosphorylation, which causes the activation of PI3K/Akt, MAPK, and PLC-γ signaling cascades [12]. Here we have found that DHF triggers autophagy induction, which is mediated through the TrkB phosphorylation and subsequent regulation of MAPK pathway. Previous studies have shown that the LKB1-AMPK signaling axis, which could be regulated by ERK1/2 [43], is a key regulator of autophagy and plays an important role in cellular metabolic sensing and regulation of cell growth, proliferation, and survival [34, 35]. We here report that DHF reduces ERK1/2 phosphorylation in MPP^+^-treated N2A cells, and consequently activates LKB1 and AMPK (Fig. 6). At present study, DHF inhibited the activation of ERK1/2, which is consistent with other studies that DHF also reduced the ERK signaling [22, 44]. Nonetheless, some contradictory results show that the ERK1/2 is activated after DHF treatment [17, 45]. One possibility is that DHF reduces ROS with its anti-oxidative property [23], which subsequently inhibited ERK1/2 activation [46].

As a mimetic of BDNF, DHF exerts similar effects as BDNF. The role of BDNF in the regulation of autophagy has been extensively studied [47]. Generally, BDNF was thought to suppress autophagy. It has been reported that the regulation of autophagy in the brain is paralleled by changes in BDNF levels, and BDNF regulates synaptic plasticity by suppressing the autophagic flux in neurons [48]. However, some studies have shown that BDNF-TrkB promotes autophagy flux [24, 25]. These discrepancies may be accounted for by different cell types and pathological conditions. In the present study, we have found DHF promotes autophagic flux in PD models. DHF specifically binds to TrkB receptor, while BDNF acts on both the p75 neurotrophic receptor and the TrkB receptor. Moreover, the tyrosine phosphorylated residues on TrkB intracellular domain and their relative abundance are different after BDNF and DHF stimulations, which may exert different downstream effects [18]. Given the different characteristics of DHF and BDNF, determining the different roles and potential mechanisms of DHF and BDNF in the regulation of autophagy is essential in future research.

In conclusion, this study shows that DHF alleviates motor deficits and reduces the loss of dopaminergic neurons in MPTP-induced mouse model of PD. These protective effects are involved in the induction of autophagy by increased phosphorylation of AMPK via ERK1/2- regulated LKB1 activation. These findings indicate that potential therapeutic capabilities of DHF to slow the progression of PD by improving impaired autophagy.

## Materials and methods

### Animals

C57BL/6 male mice (25–30 g) were purchased from Charles River Laboratories and raised at the animal care center of Children’s Hospital affiliated to Chongqing Medical University. Animals were housed at room temperature in plastic cages with free access to food and water under a 12 h/12 h light/dark cycle. The mice were randomly divided into 5 groups for different treatment. All animal experiments were conducted in accordance with the Chongqing Science and Technology Commission guidelines and approved by the Chongqing Medical University Animal Care Committee, and every effort was made to minimize both animal suffering and number of animals used.

### Drugs and treatments

MPTP, MPP^+^, 3-MA, ANA-12, and U73122 were purchased from Sigma-Aldrich. Chloroquine was purchased from MCE. U0126 was purchased from Abcam. DHF was purchased from Tokyo Chemical Industry. MPTP and chloroquine were dissolved in sterile saline. DHF was dissolved in sterile saline containing 10% ethanol. MPTP groups were administrated with MPTP (30 mg/kg, i.p.) once a day for 5 days. DHF (5 mg/kg, i.p.) and chloroquine (50 mg/kg, i.p.) were injected once a day during the whole process of experiments. The control groups received the same volume of sterile saline with or without 10% ethanol.

### Antibodies

Following primary antibodies were used in the present study: anti-GAPDH (1:5000; arigo, ARG10112), anti-TH (1:1000; BD biosciences, 612300), anti-α-synuclein (1:1000; BD bioscicences, 610786), anti-p-TrkB (1:1000; abcam, ab229908), anti-trkB (1:1000; Cell Signaling Technology, 4603 T), anti-LC3A/B (1:1000; Cell Signaling Technology, 12741S), anti-P62 (1:1000; proteintech, 18420-1-AP), anti-beclin-1 (1:1000; Cell Signaling Technology, 3738S), anti-p-ERK1/2 (1:1000; Cell Signaling Technology, 4370S), anti-ERK1/2 (1:1000; Cell Signaling Technology, 4695), anti-p-LKB1 (1:1000; Cell Signaling Technology, 3482S), anti-LKB1 (1:1000; Cell Signaling Technology, 3047), anti-p-AMPK (1:1000; Cell Signaling Technology, 2535S), anti-p-mTOR (1:1000; Cell Signaling Technology, 5536T), anti-AMPK (1:1000; Cell Signaling Technology, 2532) and anti-p-ULK1 (1:1000; Cell Signaling Technology, 5869).

### Rotarod test

The rotarod test was performed to analyze motor function in PD model mice. Briefly, mice were received 4 rounds of training on the rotarod after 2-week drug administration. In the first two rounds of training, mice were trained at constant speed of 20 rpm for 3 min. In the following two rounds, mice were trained at constant speed of 20 rpm for 3 min in rocking mode, in which the rotarod changed rotation direction from clockwise to counterclockwise every 3 turns. On the second day, mice were given formal rotarod test with rotation speed at 20 rpm in rocking mode. The test consisted of 10 times with time interval of 20 min, and the time that the mouse remained on the rod during each test was recorded. Maximum test time (cut-off limit) was 300 s.

### Pole test

The pole test was used to measure the degree of bradykinesia, as previously described with some modifications [49]. In brief, mice were put upward on the top of a rough surfaced pole (length: 50 cm, diameter: 1.5 cm), and the latency to descend the pole was measured. Trials were excluded if the mouse fell or slid down the pole rather than climbed down. Each mouse was trained for 2 trials on the first day and tested for 5 trials on the second day. The lowest latency to descend the pole was analyzed.

### Wire suspension test

Wire suspension test was used to test motor function in PD model mice. The whole set contains a wire and two platforms. The wire was fixed horizontally to two platforms and was kept 80 cm long, 25 cm high. Each mouse was suspended with its paws on the middle of the wire, and the time taken to reach the platform is recorded. Mice were trained with two trials on the first day and tested for five trials on the second day. The shortest latency to reach the platform was analyzed. Trials were excluded if the mouse fell from wire or stayed still. Maximum test time (cut-off limit) was 120 s.

### Western blotting

Brain tissues or N2A cells that were obtained from Professor Weihong Song (University of British Columbia, Vancouver, Canada), were lysed on ice with lysis buffer (Keygen Biotech, KGP702), and then the extracts were centrifuged at 12,000 × *g* for 15 min at 4 °C. The supernatant was collected, and protein concentration was measured by BCA protein assay kit (Thermo Fisher Scientific, Waltham, MA, USA). Equal amount of protein samples was mixed with 5× sample buffer, boiled at 95 °C for 5 min, and separated on 10–15% sodium dodecyl sulfate-polyacrylamide gel electrophoresis (SDS-PAGE). Proteins were then transferred to polyvinylidene fluoride (PVDF) membranes (Bio-Rad, Hercules, CA, USA). Membranes were blocked with 5% nonfat milk in Tris-buffered saline containing 0.1% Tween-20 (TBST) for 1 h at room temperature, and then incubated overnight at 4 °C with primary antibody. After washing 3 times in TBST, membranes were incubated with secondary antibody for 1 h at room temperature. After another 3 washes with TBST, protein band was visualized in the Bio-Rad Imager with ECL Western blotting substrate (Pierce). Immunoblotting with anti-GAPDH was used to control equal loading and protein quality. The band intensity was quantified by the Bio-Rad Quantity One software.

### TH Staining and quantification

Mice were anesthetized with urethane (1.5 g/kg, i.p.), and then perfused with phosphate-buffered saline (PBS) followed by 4% paraformaldehyde. The brain was removed and fixed in 4 % paraformaldehyde and dehydrated in 30 % sucrose solution, then sectioned into 30 µm slices with Leica cryostat. Sections were rinsed in PBS and then incubated in 3 % H_2_O_2_ for 10 min to eliminate the activity of endogenous peroxidase. After washing 3 times in PBS, brain sections were incubated in PBS solution containing 5% BSA and 0.2% Triton X-100 for 1 h at room temperature, then incubated overnight in anti-TH (1:500 dilution) at 4 °C. Finally, a biotinylated goat anti-mouse secondary antibody (Zhongshan Golden Bridge Biotechnology, PV9000) and diaminobenzidine (Zhongshan Golden Bridge Biotechnology, ZLI-9018) were used according to the manufacturer’s instruction to detect the immunoreactivity. The quantification of TH-positive cells in the SN and TH-positive neuronal terminals in the striatum were counted at six-section intervals throughout the entire extent of SN and striatum, as previously described [23].

### Immunofluorescence and confocal microscopy

N2A cells were treated with MPP^+^ and DHF for 24 h, fixed in 4 % paraformaldehyde for 20 min. After washing 3 times with PBS, cells were permeabilized with 0.3 % Triton X-100 in PBS for 30 min and blocked with 5 % BSA for 1 h at room temperature. Then, cells were incubated with rabbit anti-LC3 antibody overnight at 4 °C followed by Alexa Fluor488 (Invitrogen, A11034) conjugated secondary antibody for 1 h at room temperature. Finally, N2A cells were counterstained with DAPI (Sigma-Aldrich, D9542) and imaged with a confocal microscope.

### Statistical analysis

All data were expressed as mean ± SEM. The differences of rotarod test were analyzed by two-way ANOVA, with treatment (group) as the between-subjects factor and test as the within-subjects factor. The data of all other experiments were analyzed by one-way ANOVA, followed by Tukey’s post hoc test. Significance level was set at *P* < 0.05.

## Supplementary information

Supplemental figure legends

Supplemental figure 1

Supplemental figure 2

## Data Availability

The datasets used and analyzed during the current study are available from the corresponding author on reasonable request.
