# Supplementary material for: 7,8-dihydroxyflavone ameliorates motor deficits via regulating autophagy in MPTP-induced mouse model of Parkinson’s disease
Source: Cell Death Discov. 2021 Sep 20;7:254. doi: 10.1038/s41420-021-00643-5 (PMC8452727; doi:10.1038/s41420-021-00643-5)
Supplement: Supplementary file 1 — Supplemental figure legends [file 41420_2021_643_MOESM1_ESM.docx]

**Supplementary figure legends**

**Fig. S1** **DHF induces ULK1 activation in MPP^+^-treated N2A cells.** (a) Sequential immunoblotting of cell lysates. (b, c) The protein levels of p-ULK1 (b) and p-mTOR (c) assessed by Western blot in MPP^+^-treated N2A cells with or without DHF treatment. Data are expressed as the mean ± SEM. **^*^**P < 0.05.

**Fig. S2 DHF-promoted autophagy is not dependent on PLC-γ-IP3-Ca^2+^ signal.** (a) Sequential immunoblotting of cell lysates. (b-d) The protein levels of p-AMPK (b), P62 (c) and LC3 (d) assessed by Western blot in MPP+-treated N2A cells with or without co-administration of DHF and U73122. Data are expressed as the mean ± SEM. **^*^**P < 0.05.
