# Supplementary figures and images for: 7,8-dihydroxyflavone ameliorates motor deficits via regulating autophagy in MPTP-induced mouse model of Parkinson’s disease
Source: Cell Death Discov. 2021 Sep 20;7:254. doi: 10.1038/s41420-021-00643-5 (PMC8452727; doi:10.1038/s41420-021-00643-5)

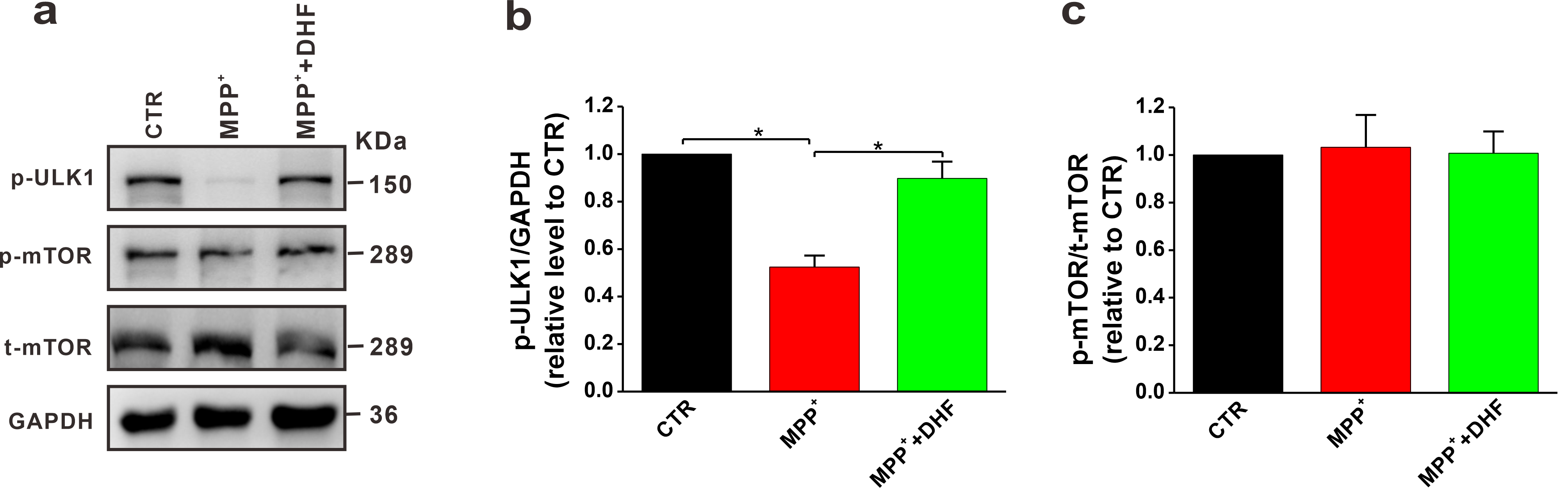

Supplement: Supplementary file 2 — Supplemental figure 1 [file 41420_2021_643_MOESM2_ESM.png]

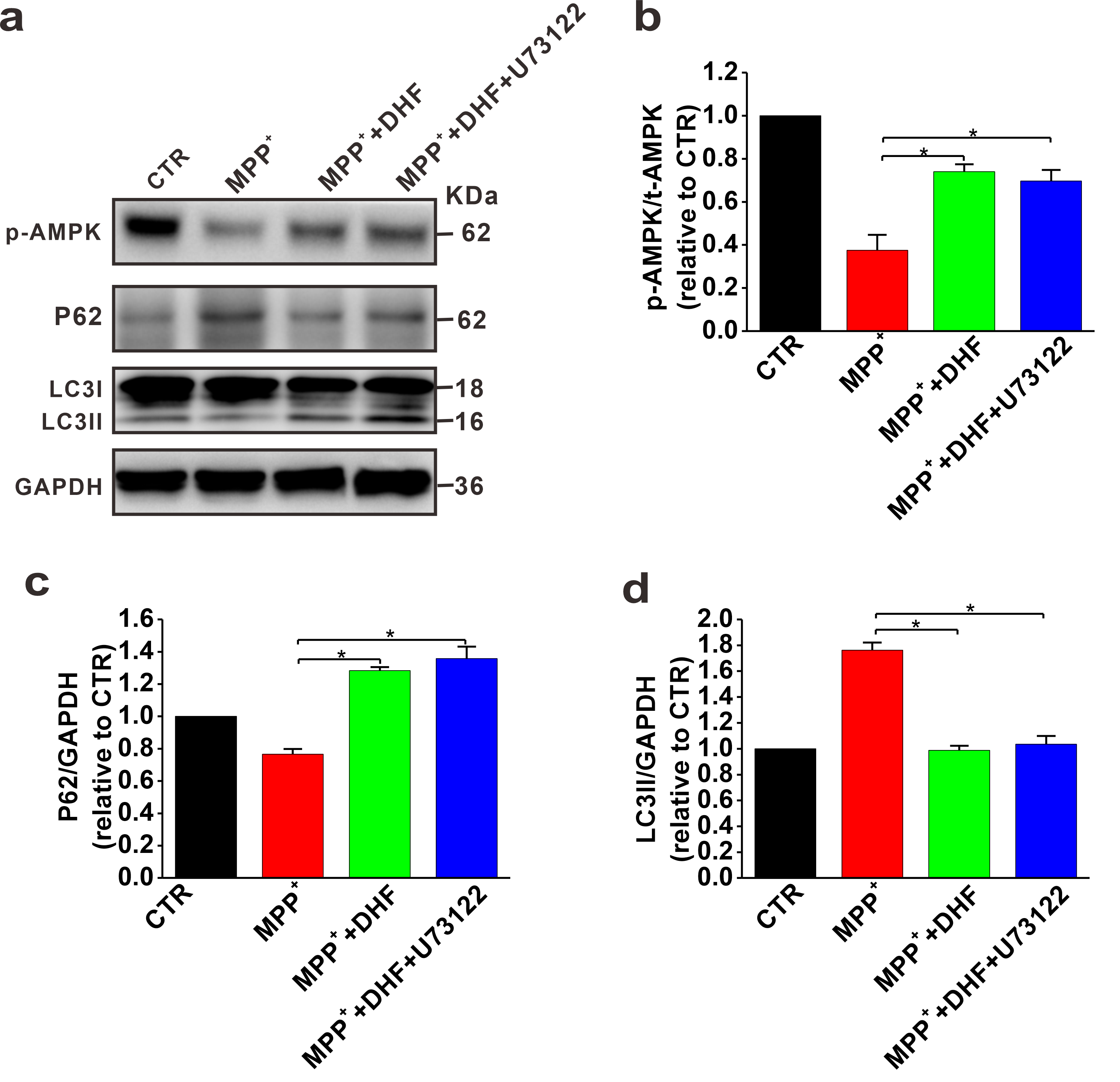

Supplement: Supplementary file 3 — Supplemental figure 2 [file 41420_2021_643_MOESM3_ESM.png]
